# Handling of the Bivalve *Pinna nobilis*, Endangered and Pathogen‐Affected Species, for Controlled Reproduction: Precautions Taken

**DOI:** 10.1002/ece3.70565

**Published:** 2024-12-15

**Authors:** Maria Paola Ferranti, Ilenia Azzena, Edoardo Batistini, Daniela Caracciolo, Marco Casu, Mariachiara Chiantore, Saul Ciriaco, Valerio Firpo, Luca Intini, Chiara Locci, Monica Montefalcone, Alice Oprandi, Daria Sanna, Fabio Scarpa, Marco Segarich

**Affiliations:** ^1^ Department of Earth, Environment and Life Sciences (DiSTAV) University of Genoa Genova Italy; ^2^ National Biodiversity Future Center (NBFC) Palermo Italy; ^3^ Department of Veterinary Medicine University of Sassari Sassari Italy; ^4^ Shoreline Società Cooperativa Trieste Italy; ^5^ Area Marina Protetta di Miramare Trieste Italy; ^6^ ARPAL, Regional Agency for the Environmental Protection Liguria Genova Italy; ^7^ Department of Biomedical Sciences University of Sassari Sassari Italy

**Keywords:** controlled reproduction, fan mussel, *Haplosporidium pinnae*, larvae, Pinnidae, transport

## Abstract

Following the increased mass mortality of *Pinna nobilis* populations in the Mediterranean, reliable protocols for the transport, maintenance, and controlled reproduction of this highly endangered species were drawn up within the European Life Pinna project. To test these protocols, the large Pinnidae *Atrina fragilis* , which shares similar habits to *P. nobilis* , has been used. In December 2022, a transport trial of nine specimens of *A. fragilis* from Trieste (NE Italy) to Camogli (NW Italy) was carried out. Two positioning (vertical and horizontal) of the specimens were tested inside the transport box. In the laboratory, after acclimatization, the specimens were placed inside three tanks and fed three times a week with a mix of live microalgae and artificial feed. The transport and maintenance protocols tested on *A. fragilis* were then applied to 11 *P. nobilis* collected in the Venice lagoon (NE Italy) and transported to the laboratory in June 2023. Due to the possible infection with *Haplosporidium pinnae*, considered one of the main etiological agents of mass mortality, *P. nobilis* individuals were evaluated through molecular analyses during their stay in the tank. Furthermore, these specimens were used as breeders: They spontaneously released already fertilized eggs, as a consequence of transport stress. Rapid larval development stopped at the early veliger stage, and the larvae were fed three times a week with a mixture of microalgae. After the reproduction period, the four specimens that survived 6 months in the laboratory, and constantly tested negative for *H. pinnae*, were transplanted to the Capo Mortola Marine Protected Area (Liguria, Italy) and monitored monthly.

## Graphical abstract

Reliable protocols have been developed for the transport, maintenance, and controlled reproduction of *Pinna nobilis*, a highly endangered species. *P. nobilis* individuals were evaluated through molecular analyses during their stay in the laboratory tanks. In the laboratory, the specimens were fed three times a week with a mix of live microalgae and artificial feed. Furthermore, these specimens were used as breeders, and they spontaneously released already fertilized eggs, post transport stress. Rapid larval development was observed and followed, until D‐larva stage.

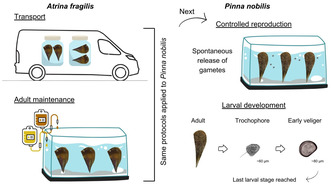

## 1 Introduction

The fan mussel *Pinna nobilis* Linnaeus, 1758 is an endemic species to the Mediterranean Sea, with important ecological roles. As an ecosystem engineer, in a soft bottom surrounding, it provides a suitable habitat for hard substrate colonizers (Katsanevakis et al. 2022). Its ability to filter large amounts of water favors organic matter depletion and turbidity reduction (Trigos et al. 2014). Being the largest bivalve in the Mediterranean, it also contributes significantly to carbon sequestration, as is the case for several species of mollusk (Tang et al. 2018). Their feeding activities remove a significant amount of particulate organic carbon from seawater, sequestering carbon as CaCO_3_ in their shells and soft tissues (Zhang et al. 2021; Gu et al. 2022). Since ancient times, fan mussels have been exploited not only for human consumption but also to produce the so‐called “sea silk” from the byssus for several handicrafts. Exploitation and habitat degradation (Basso et al. 2015) led to the decline of *P. nobilis* populations. For these reasons, the species was listed as endangered in Annex II of the Barcelona Convention and in Annex IV of the Habitat Directive 92/43/EEC. Protection was effective, but since 2016, this species has been affected by a mass outbreak caused by a multifactorial disease (Scarpa et al. 2020; Carella, Palić et al. 2023; Theodorou et al. 2024), related to the possible combined action of different pathogens whose specific effect remains unclear: Protozoans such as *Haplosporidium pinnae* (haplosporidian protozoan; Catanese et al. 2018; Panarese et al. 2019; Katsanevakis 2019; Lattos et al. 2023) and *Perkinsus* spp. (Carella et al. 2020), bacteria such as *Mycobacterium* sp. (Carella et al. 2019) and *Vibrio* spp. (Andree et al. 2020; Prado, Carrasco et al. 2020; Lattos, Bitchava et al. 2021), and viruses such as piconavirus (Carella, Prado et al. 2023) that are associated to the drastic reduction of its populations (Lattos et al. 2020; Lattos, Bitchava et al. 2021; Lattos, Feidantsis et al. 2021; Vázquez‐Luis et al. 2021; Katsanevakis et al. 2022). In this context, other pinnids appear to be resistant to pathogen infection and possibly benefit from *P. nobilis* reduction, as evidenced by the recently observed range expansion of *Pinna rudis* (Zotou et al. 2023; Oprandi et al. 2024; Rubino, Fanelli, and Denti 2024). This phenomenon led to change the status of *P. nobilis* from “vulnerable” to “critically endangered” in the IUCN Red List of threatened species. Due to the critical situation of *P. nobilis* populations throughout the Mediterranean, different actions have been considered to reverse their collapse. These include translocation of adult and juvenile individuals to safer areas (e.g., pathogen‐free areas, according to sentinel species/*Pinna* monitoring for protozoans or lagoons, where environmental conditions seem to be more suitable for species survival), captive breeding (Kersting et al. 2019; Hernandis, Ibarrola et al. 2023; Hernandis, Prado et al. 2023), and research projects on the conservation of *P. nobilis* . These include the European Life Pinna project “Conservation and re‐stocking of *Pinna nobilis* in the Western Mediterranean and Adriatic Sea” (LIFE20 NAT/IT/001122), as well as of other interconnected projects, such as Life Pinnarca (LIFE20‐NAT/ES/001265), Restorfan Medpan—Small Project (2019–2020), and Marmara'nin umudu Pina.

Maintenance in a controlled environment could enhance the survival of specimens, but so far, the long‐term maintenance of *P. nobilis* is not easily performed due to mortality from both *Haplosporidium pinnae*, *Vibrio mediterranei* and *Vibrio splendidus* (García‐March et al. 2020; Prado, Cabanes et al. 2020; Prado, Carrasco et al. 2020; Hernandis 2021; Lattos, Bitchava et al. 2021). Different trials have already been performed to transport adult pinnids in laboratory/aquarium tanks (e.g., Prado, Cabanes et al. 2020; Hernandis et al. 2021), and maintenance of both adult and juvenile specimens of different Pinnidae species in a controlled environment has been performed (Ford and Borrero 2001; Prado et al. 2020; Hernandis 2021). *Pinna nobilis* is a successive hermaphrodite, with asynchronous gamete maturation. Its reproductive cycle is known in different Mediterranean areas (Butler, Vicente, and de Gaulejac 1993; De Gaulejac 1993, 1995; Basso et al. 2015; Deudero et al. 2017; Acarli et al. 2018; Acarli 2021), yet few studies exist regarding spawning induction and artificial fertilization, and unfortunately none achieved larval settlement and production of juveniles (Trigos et al. 2018; Hernandis, Ibarrola et al. 2023; Hernandis, Prado et al. 2023). The present study, carried out within the Life Pinna project, reports reliable protocols for transport, maintenance, controlled reproduction, and early larval stages' maintenance of *Pinna nobilis* to establish a sustainable and effective approach to the restoration of the species in the Mediterranean.

## 2 Methods

Preliminary trials were performed on the closely related but not endangered species *Atrina fragilis* (Pennant 1777) that was used as a model species for transport, maintenance, and spawning induction. This species is a large Pinnidae, which shares similar habits to *P. nobilis* such as living in mud, sand, gravel, and seagrass meadows (Poutiers 1987). It is widely distributed in the Atlantic and the Mediterranean, although it is locally considered rare (Fryganiotis, Antoniadou, and Chintiroglou 2013). *A. fragilis* is not a protected species, except in the UK, where it has been assigned the status of priority species for conservation (Solandt 2003; Hiscock and Jones 2004), as it is considered endangered (Act n. 1990–54) mainly due to habitat degradation and intensive bottom trawling (Solandt 2003; Fryganiotis, Antoniadou, and Chintiroglou 2013). *A. fragilis* transport and maintenance were performed in autumn 2022, and the protocols were applied on *P. nobilis* in summer 2023.

### 2.1 Sampling and Transport of *A. fragilis* and *P. nobilis*

**FIGURE 1:**
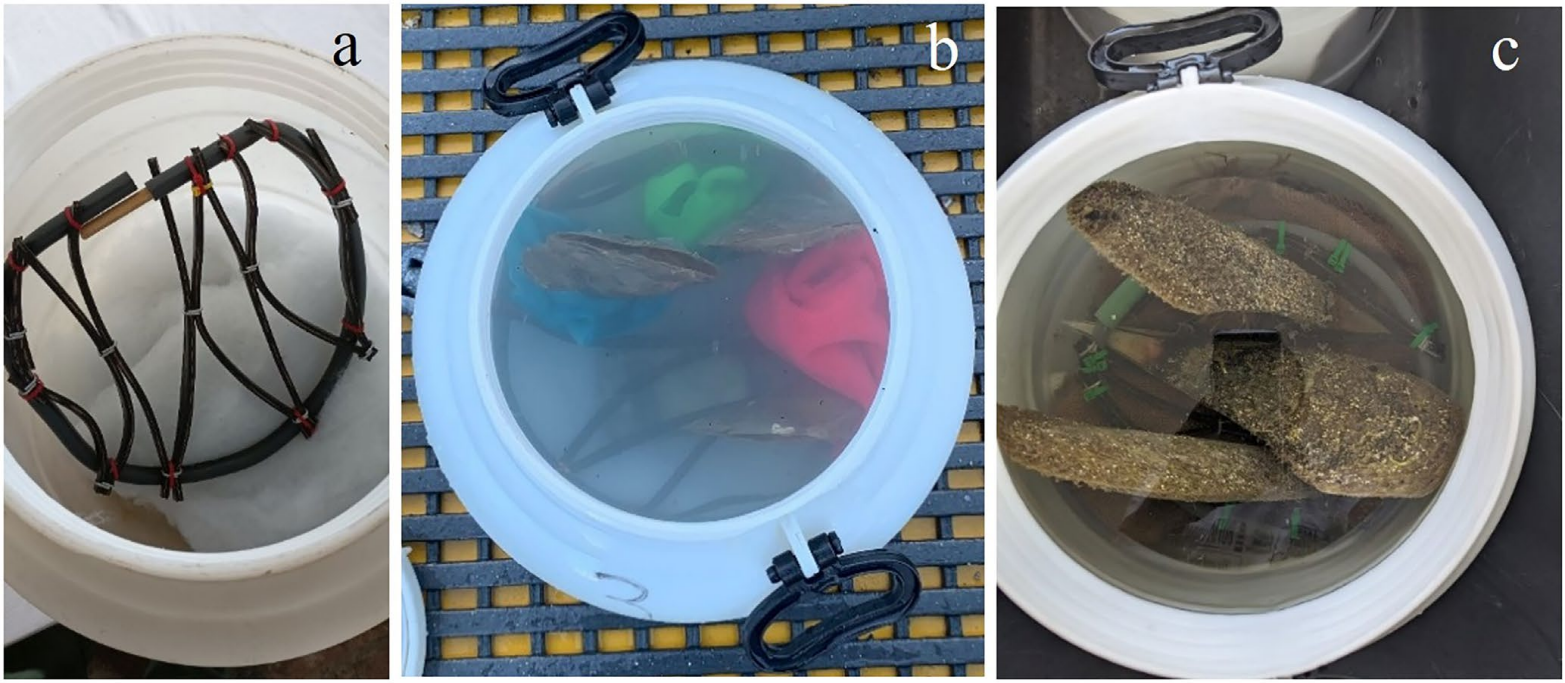
Vertical system. (a) Structure with rubber net; (b) *Atrina fragilis* specimens into the transport box; (c) *Pinna nobilis* specimens into the transport box.

On 1 December 2022, nine specimens of *A. fragilis* were collected in the Gulf of Trieste, near the buffer zone of the Miramare Marine Protected Area (MPA), at a depth of 15 m on a muddy bottom. The specimens included seven adults, with the shell height ranging from 20 cm to 24 cm, and two juveniles with a height of 6.3–8.3 cm. The specimens were transported to the CNR‐IBF laboratory in Camogli (Liguria, NE Italy), about 30 km from Genova. The specimens were placed inside food‐grade plastic containers (30 L) and transported in a refrigerated van, with temperature control capabilities set at 18°C for an 8 h journey. During the travel, the condition of the individuals, the containers, and bubblers' efficiency was checked every 2 h. Prior to transport, the containers were filled with water, and the organisms were carefully placed in water, ensuring they remained fully submerged at all times. Then, the specimens were secured and wrapped with a soft material (cloth, rock wool, jute, etc.) (Figure 1b,c). Aeration inside the containers was ensured by a bubbler device (AMTRA, 5 V, 360 L/h). The aeration system was assembled using a tube whith an air stone to oxygenate the box. A wad of rock wool was placed in the upper part of the tube in order to prevent the formation of large bubbles that could damage the organisms' filtering system.

Two different transport methods were tested:

1. Vertical system (Figure 1): A 10 cm thick layer of rock wool was placed at the bottom of the box. A rubber structure was then placed on top of the rock wool to keep the individuals in a vertical position. The structure consisted of a rubber net attached to a frame made of plastic tubes (Figure 1a). The system kept the individuals in a vertical position while allowing the valves to open.
2. Horizontal system: The individuals were laid horizontally on the rock wool at the bottom of the box, without any special structure for fixing.

On 19 June 2023, 11 adult specimens of *P. nobilis* were collected in Venice Lagoon at a depth of 4 m (water parameters: 23.2°C temperature, 36.22 PSU salinity, and 7.69 pH). The shell height of the specimens ranged from 28 to 43.5 cm. The transport to the Camogli laboratory lasted 6 h. The specimens were transported by means of a refrigerated van, with temperature control capabilities set at 18°C, using the vertical system tested on *A. fragilis* (Figure 1c).

### 2.2 Maintenance in the Laboratory of *A. fragilis* and *P. nobilis*

In the laboratory, the specimens of both species were acclimatized, and the procedure lasted around 1 h, with a water change rate of 3 L of water changed every 10 min inside the 30 L boxes used for the transport.

**FIGURE 2:**
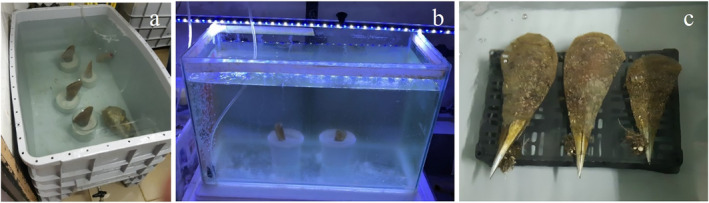
Accommodation of Pinnidae specimens in laboratory tanks. (a) *A. fragilis* adults; (b) *A. fragilis* juveniles; (c) *P. nobilis* adults.

For *A. fragilis* , the seven adult specimens were placed inside two 240 L tanks and the two juveniles in a 40 L aquarium. In the laboratory tanks, all specimens (adults and juveniles) were placed vertically inside glass containers filled with sand (Figure 2a,b).

The 11 adult *P. nobilis* specimens, were placed inside two 240 L tanks (three specimens/tank) and one 540 L tank (five specimens). Inside the tanks, all specimens were placed horizontally on plastic boxes (Figure 2c) to facilitate their handling for spawning induction trials in order to achieve controlled reproduction. This positioning is also in agreement with the study carried out by Hernandis et al. (2022) who did not observe significant differences in the physiology of Pinnids between the laying down and standing positions.

For both species, being a system without recirculation, the tanks were cleaned three times a week to remove feces and any food residual, and partial water change (i.e., 2/3 of the total volume, three times a week) was performed to avoid air exposure of the specimens. The sea water used for the tanks was filtered through a sand filter to remove large particles; then, the finest fraction was removed with a microfiltration system composed by two cartridge filters (10 μm and 1 μm), and, at the end of the process, water was sterilized through three UV lamps in series (Amtra Scudo Inox UVC‐36w; Ford and Borrero 2001; Prado, Cabanes et al. 2020; Hernandis 2021). For *P. nobilis* specimens, management of the water change was more complex as the wastewater was stored in separate tanks and chemically sterilized (0.4 mL/L sodium hypochlorite with aeration for 24 h and then 0.024 g/L sodium thiosulfate to eliminate excess chlorine) to avoid discharging potentially pathogen‐contaminated water into the sea.

Air was provided in the tanks through air stones connected to a compressor, and the photoperiod was regulated using an LED lamp (12 h light/12 h dark).

All specimens were fed three times a week with a mix of live microalgae ( *Isochrysis galbana* : At an average concentration of 3.25 × 10^6^ cell/mL; *Chaetoceros calcitrans*: At an average concentration of 3.75 × 10^6^ cell/mL; *Diacronema lutheri*: At an average concentration of 2.33 × 10^6^ cell/mL) and an aliquot of Easy Reef artificial feed (Table 1). The microalgae are grown in the laboratory inside 40 L bioreactors, at a salinity between 31.5–32 practical salinity units (PSU). The density of cultured algal cells was calculated using the Bürker hemocytometer. The dose of microalgae to be supplied to specimens of both species was calculated taking into account information from different studies (Trigos et al. 2015; Prado, Cabanes et al. 2020; Hernandis et al. 2022). On the basis of the specimen size and algal culture density used (see concentrations above), *A. fragilis* adult specimens (on average 22.14 cm in shell height) were fed providing for each individual 200 mL of each microalgal species/day (for the total 600 mL of microalgal mix for individual *Atrina* specimen), and juvenile specimens (on average 7.30 cm in shell height) were fed, individually, with 45 mL of each microalgal species/day (for the total 135 mL of microalgal mix for individual *Atrina* sjuvenile). *P. nobilis* specimens (all adults, on average 37.85 cm in height) were fed 350 mL of each microalgal species/day (for the total 1050 mL of microalgal mix for individual *Pinna* specimen) (Table 1).

**TABLE 1:** Daily diet of *Atrina fragilis* and *Pinna nobilis* specimens maintained in the laboratory (average algal cell density: *Isochrysis galbana* : 3.25 × 10^6^ cell/mL, *Chaetoceros calcitrans*: 3.75 × 10^6^ cell/mL, *Diacronema lutheri*: 2.33 × 10^6^ cell/mL).

Tanks	Diet	*Atrina fragilis* (mL/specimen/day)	*Pinna nobilis* (mL/specimen/day)
Adults	*Isochrysis galbana* *Diacronema lutheri* *Chaetoceros calcitrans* + Easy Reef	200 mL 200 mL 200 mL + 4 mL/250 L of tank water/day	350 mL 350 mL 350 mL + 4 mL/250 L of tank water/day
Juveniles	*Isochrysis galbana* *Diacronema lutheri* *Chaetoceros calcitrans* + Easy Reef	45 mL 45 mL 45 mL + 4 mL/250 L of tank water/day	—

While the feeding ration of living microalgae was provided per individual pinnid specimen (independenly of the size of the tank), the artificial feed was provided according to the tank size (40 L tank: 0.65 mL/day; 240 L tanks: 4 mL/day; 540 L tank: 9 mL/day), following the instructions of the product (4 mL/day for every 250 L of tank water).

### 2.3 Spawning Induction and Fertilization of *A. fragilis* and *P. nobilis*

Spawning induction trials with thermal shock were carried out on *A. fragilis* after 5 months since their arrival in the laboratory (Trigos et al. 2018; Hernandis, Ibarrola et al. 2023).

*A. fragilis* specimens were stimulated using the thermal shock method: They were maintained at ambient temperature and moved repeatedly (about every 50 min) from cold water tanks to warmer ones, with a thermal gap of 10°C (+ and –5°C from room temperature), over a period of 3–6 h (Trigos et al. 2018; Hernandis, Prado et al. 2023). In May 2023, three spawning induction trials were carried out, and the treatment lasted from 3 to 6 h. After being submitted to the induction stimulus, specimens were placed in tanks filled with seawater at room temperature and observed to detect spawning for a few hours. Once the stimulation was concluded, the specimens were placed back in their tanks. No spawning was obtained.

In the case of *P. nobilis* , the spawning occurred naturally in the summer of 2023, without the need for thermal induction, as the stress of collection and transport triggered the release of gametes. The release of both male gametes and already fertilized eggs (self‐fertilization) has been observed (Hernandis, Prado et al. 2023). These spawning events will be indicated as Pn_F1, Pn_F2, and Pn_F3. The eggs released were collected and placed in glass beakers. The number of eggs was counted by homogenizing the sample in a known volume of seawater, taking a 1 mL subsample and counting in a Sedgwick Rafter Counting Chamber. The development of embryo was monitored, following their rapid development until the formation of the larvae (Figure 5; Table 3). Then, the zygotes were introduced in various tanks/aquaria, with different volumes (30, 60, 83, and 240 L).

### 2.4 Rearing of *P. nobilis* Larvae

The larvae were reared at a water temperature of about 25°C (room temperature) and 37.5 PSU of salinity; the water used was filtered and sterilized in the same way as for adult specimens, but for the first larval stages, antibiotic was also added (Penicillin G sodium salt, 10 mg/L and Streptomycin sulfate salt, 20 mg/L, Sigma Aldrich, according to Huggett et al. 2005) to prevent bacterial proliferation in larval cultures. The larvae were placed in tanks of different volumes at the density of 7–50 larvae/mL. Then, the larvae were counted daily by homogenizing the sample in a known seawater volume, taking a 1 mL subsample, and counting in a Sedgwick Rafter Counting Chamber; at the same time, the stage of larval development was monitored.

The water in the larval tanks was changed three times a week by filtering the water through a 50 μm filter that was partially submerged inside the tank, so that the larvae were not left dry on the filter. Then, the larvae were put back into the tanks filled with renewed water and fed. For safety, the wastewater from the larvae tanks, as well as the adults, was stored in separate tanks and chemically sterilized. As for the larvae, the sterilization of the wastewater was stopped when molecular analyses revealed that the larvae were negative for *H. pinnae*. Daily, the larvae were fed a fixed dose of a mix of the three microalgae ( *I. galbana* , *C. calcitrans* , and *D. lutheri* ). Based on the larval density and tank volume, a total of approximately 250 or 750 mL of the microalgae mix was provided daily (about 85 mL or 250 mL for each algal species/tank/day), corresponding to an average of 5 × 10^−4^ mL of microalgal mix/day/larva (1.5 × 10^3^ cells/larva for the algal mix).

### 2.5 Molecular Diagnostic Analyses of Pathogens of *P. nobilis*

Filters from the three tanks housing captive *P. nobilis* individuals were analyzed using molecular diagnostic analyses to detect pathogens. These analyses focused their attention on the presence of protozoans belonging to the genus *Haplosporidium* with particular reference to the species *H. pinnae* that is considered one of the main etiological agents for the mass mortality of *P. nobilis* (Vázquez‐Luis et al. 2017; Darriba 2017; Catanese et al. 2018). For each filter, DNA extractions were performed in triplicate from both the proximal and central regions. The extracted DNA from each sample was analyzed by standard PCR and quantitative PCR (qPCR) to identify the possible presence of the protozoan *H. pinnae*. The same analyses were also performed on both *P. nobilis* individuals inside the tanks and their larvae. DNA extraction from alive fan mussels utilized a small mantle tissue fragment collected by means of a nonlethal sampling method, as previously standardized by our research group (see Sanna et al. 2013 for details). This method ensured minimal damage to the shells and soft tissues of *P. nobilis* . For dead individuals, samples from all organs were collected and analyzed. As for the larvae, some of the specimens which died a few days after hatching were used for the diagnostic analyses.

#### 2.5.1 Molecular Diagnostic Analysis

Genomic DNA extraction was performed on fragments of mantle tissue from 11 living individuals, their dead larvae, and the tanks' filters. Total genomic DNA was isolated using the Macherey‐Nagel Nucleo Spin Tissue Kit (MACHEREY‐NAGEL GmbH & Co. KG; Neumann Neander Str. 6–8 D‐52355 Düren, Germany) following the manufacturer's instructions. DNA solutions were quantified using a Nanodrop Lite spectrophotometer (by Thermo Scientific;Waltham, MA, USA), which showed an average yield of 50 ng/μL. All samples were screened for pathogen DNA using both standard PCR and qPCR with primers specific for protozoan ribosomal 18S regions (Catanese et al. 2018; Poutierst et al. 2000). For *P. nobilis* specimens, this screening was periodically repeated for a constant monitoring of pathogens’ infection.

PCRs were conducted in a total volume of 25 μL. On average, 10 ng of the total genome was combined with 0.6 μM of each primer and one pellet of PuReTaq Ready‐To‐Go PCR beads (GE Healthcare; 9900 West Innovation Drive, Wauwatosa, WI, USA), containing stabilizers, bovine serum albumin (BSA), deoxynucleotide triphosphates, 2.5 units of PuReTaq DNA polymerase, and reaction buffer. Each reconstituted bead to a final volume of 25 μL had a concentration of 200 μM for each dNTP and 1.5 mM MgCl_2_. PCRs were performed in a GeneAmp PCR System 9700 Thermal Cycler (Applied Biosystems; 81 Wyman Street, Waltham, MA, USA) with the following program: 1 cycle of 4 min at 94°C, 35 cycles of 30 s at 94°C, 30 s at 54°C, and 30 s at 72°C. A post‐treatment of 5 min at 72°C and a final cooling at 4°C were carried out. Both positive (old samples stored in our laboratory that produces good PCR products for the primers used in this study) and negative controls were used to assess the effectiveness of the PCR protocols and confirm the absence of potential inhibitors.

The qPCR assay for detecting and quantifying the occurrence of *H. pinnae* in the samples was performed by duplicate analyses using the primer pair HpF1/HpR2, (Renault et al. 2000) on a StepOne real‐time PCR system (Applied Biosystems). Amplification reactions were carried out in a total volume of 8 μL, including 8 ng of genomic DNA (100 ng), 4 μL of SYBR Green Master Mix (Applied Biosystems), 0.17 μL of each specific primer at a concentration of 10 μM (0,0017 nM), and adjusted to 8 μL with distilled water.

Negative controls and positive controls (samples with *H. pinnae* infection confirmed by both standard PCR and qPCR amplification) were included in each qPCR assay. After testing various protocol temperatures, the final qPCR program was set as follows: 1 cycle for 2 min and 30 s at 95°C; 40 amplification cycles at 95°C for 15 s, 57°C for 22 s, and 72°C for 23 s. Standard curves were calculated using serial dilutions of *P. nobilis* samples previously confirmed positive by standard PCR and sequencing. The efficiency (E) was determined from the slope of the standard curve using the formula E = 10^(−1/slope) – 1. A melting curve was generated at the end of the amplification process with temperature increments of 1°C/s, starting at 52°C and ending at 95°C, to ensure amplification of a single qPCR product for the primers. Amplifications were also validated by electrophoresis on 2% agarose gels in 1× TAE (Tris acetate EDTA buffer), stained with Red Nucleic Acid Stain (Biotium Inc. Fremont, CA, USA).

Molecular diagnostic analyses began approximately 1 month after the 11 individuals of *P. nobilis* were placed in the tanks and were repeated twice after and 30 days’ interval to ensure a continuous assessment of the presence of *H. pinnae* in the tissues of fan mussels.

## 3 Results

### 3.1 Survival Rate of *A. fragilis* and *P. nobilis*

**FIGURE 3:**
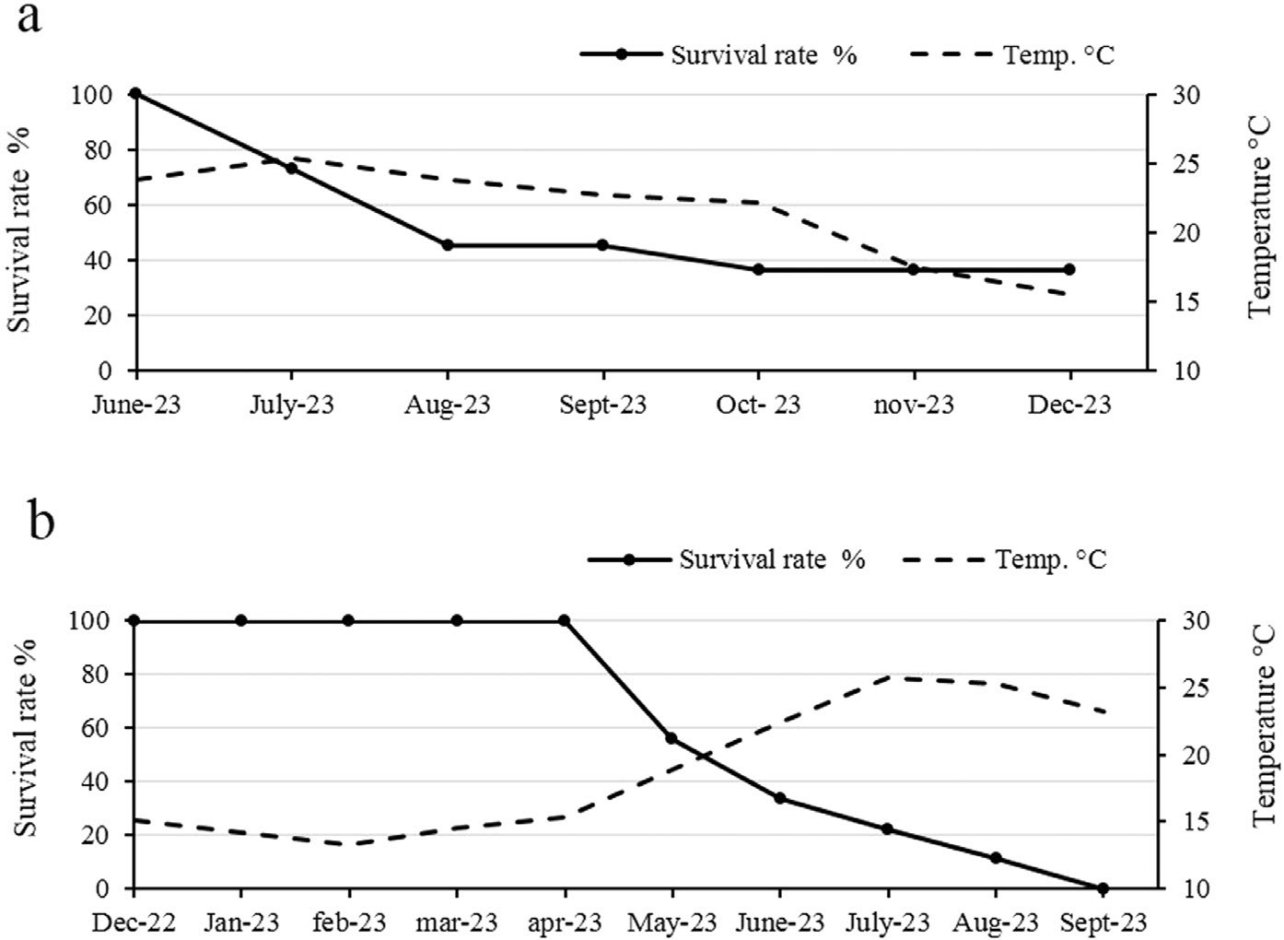
Survival rate. (a) *Atrina fragilis* specimens (adults and juveniles); (b) *Pinna nobilis* specimens.

Within 9 months, since their arrival in the laboratory, eight out of nine *A. fragilis* specimens died, with the total survival rate of 11% (Figure 3a). The juveniles survived until September, while the adults only survived until June, when the last surviving adult specimen was returned to the sea in the Gulf of Trieste (Figure 3a).

As for as the *P. nobilis* specimens, about 6 months after transport to the laboratory, 4 out of the 11 specimens survived, with a survival rate of 36.4% (Figure 3b).

The dead *P. nobilis* specimens were subjected to diagnostic molecular analysis to determine the eventual presence of *H. pinnae*. All specimens resulted positive for *H. pinnae*. The four adult specimens that survived and were negative for *H. pinnae* were translocated to the marine protected area of Capo Mortola (Liguria, Italy) on 14 December 2023, in order to guarantee gonad maturation in the natural environment, and are monitored monthly.

Size measurement was performed twice a month for juveniles of *A. fragilis* , without observing significant growth. For adults of both species, measurements were taken at the beginning and end of the laboratory maintenance period, but no shell growth was observed. No significant effect of size on mortality was detected: Surviving *A. fragilis* was 23 cm in shell height, while dead ones ranged 20–24 cm; surviving *P. nobilis* was 28–40 cm in shell height, while dead ones ranged 34–43.5 cm.

### 3.2 Spawning and Ferilization of *A. fragilis* and *P. nobilis*

In May 2023, *A. fragilis* adult specimens were subjected to three spawning induction trials by means of a thermal shock with a temperature change of approximately Δ = 10°C (Trigos et al. 2018), without obtaining any gamete release.

**FIGURE 4:**
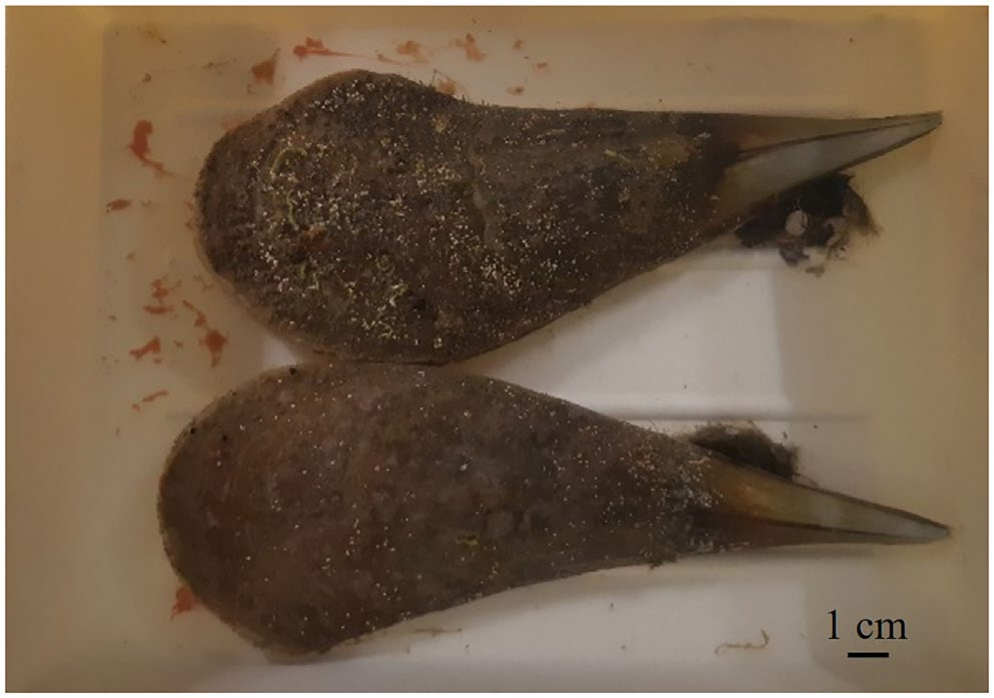
Spawning of *Pinna nobilis* specimens, releasing already fertilized eggs. Line bar: 1 cm.

**FIGURE 5:**
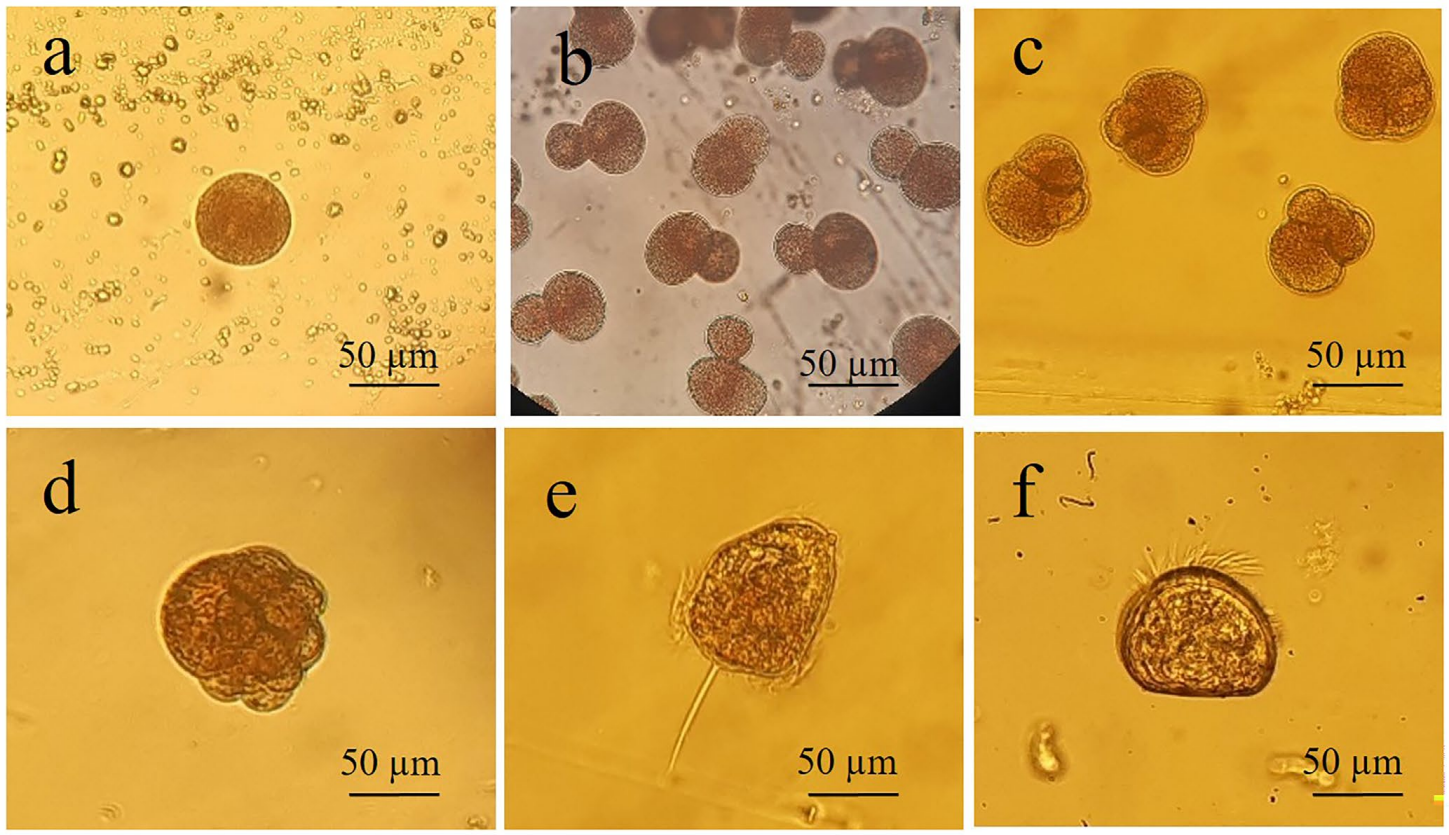
Larval developmental stages of Pinna nobilis. (a) Egg with polar body; (b) first complete division; (c) third and fourth divisions; (d) end of cell division phase (> 8 cells); (e) € late trochophore; (f) early veliger. Line bar: 50 μm.

Instead for *P. nobilis* adult specimens, between June and July 2023, five spontaneous gamete releases occurred, stimulated by transport stress (according to the definition of transport stress by Hernandis et al. 2022 that encompasses 15 days from the transport event), but only three of these resulted in fertilization. Among the 11 specimens, 6 specimens released only male gametes (M:“male”), 3 specimens released already fertilized eggs (F:“female; Figure 4), and in 2 specimens, we could not observe as to whether they had released male or female gametes (I: indeterminate). For simplicity, both in the text and in Table 2, we have preferred to use the terms male, female, and indeterminate, only to distinguish the specimens on the basis of the gametes released in the three spawning events. The released eggs, already fertilized, were collected, counted, and the rapid development of the zygote to the formation of the larva was observed (Tables 2, 3; Figure 5). Among the nine specimens that released gametes, five tested positive for *H. pinnae*, and four were negative. Furthermore, the positive specimens released both already fertilized eggs and sperm.

**TABLE 2:** Data relating to spontaneous spawning events of *Pinna nobilis* (*specimens tested positive for *Haplosporidium pinnae*; M: Male; F: Female; I: Indeterminate).

Date	Spawning (n° specimens: Females, males, and indeterminate)	ID specimens	ID fertilization	N° eggs release	N° fertilized eggs	Fertilization rate	Trochophore rate
20/06/2023	5 (3F; 2 M)	F: 4*; 5*; 6* M: 1, 2, 3, 7; 9*	Pn_F1	11,310.000	10,780.000	95.31%	99.6%
21/06/2023	5 (3F; 2 M)	F: 4*; 5*; 6* M: 7; 9*	—	Eggs not counted	—	—	—
22/06/2023	2 M	M: 7; 10*	—	—	—	—	—
28/06/2023	3F	F: 4*; 5*; 6*	Pn_F2	3313.800	3280.200	99%	94.5%
05/07/2023	5 (3 M; 2I)	M: 7; 9*; 10* I: 8*; 11*	Pn_F3	Eggs not counted	—	—	—
Total				14,623.800	14,060.200		

**TABLE 3:** Larval development timeline in *Pinna nobilis* at around 25°C, in comparison with Trigos et al. 2018 (21°C). NM: Not measured.

Stage	Cumulated time (h:min)			Size (μm)	
	This study		Trigos et al. 2018	This study	Trigos et al. 2018
	Pn_F1	Pn_F2			
Spawning/fertilization	0:00	0:00	0:00	50	50
Eggs with polar body	NM		0:15	50	50
Double membrane	NM		0:30	50	50
1st inclusion	NM		0:40	—	55
1st complete division	00:25	00:22	01:00	—	—
3th and 4th division	01:15	00:30	03:00	53	—
End of cell division phase (> 8 cells)	01:50	02:10	04:30		—
Blastule	—	—	05.00	—	55
Gastrule	—	—	08:00	—	55
Early trochophore	05:55	04:41	22:00	—	65
Late trochophore	20:50	—	30:00	65	70
Early veliger	22:36	26:30	48:00	75–80	85
Late veliger	—	—	72:00	—	90
Early umbonate	—	—	144:00	—	100
Pediveliger	—	—	168:00	—	110

Only the first two fertilizations (Pn_F1 and Pn_F2) were followed completely; the eggs of the third fertilization were not counted but added directly into the tanks of the second fertilization. A total of 14.623.800 eggs were released, with high fertilization rates (between 95.31% and 99%, resulting in 14.060.200 fertilized eggs) and a subsequent high trochophore rate (between 94.5% and 99.6%).

### 3.3 Larval Rearing of *P. nobilis*

The different developmental stages of the zygote up to the formation of the trochophore and veliger were observed, along the larval maintenance period which stopped at 9 days post‐fertilization (dpf) for both fertilizations (Pn_F1 and Pn_F2) Larval development was very rapid; approximately 22 and 26 h post‐fertilization (hpf), we observed the initial veliger stage and Pn_F1 and Pn_F2 fertilization, respectively (Figure 5f; Table 3) This rapid development was probably due to the high water temperature (around 25°C) because cultures could not be maintained in temperature‐controlled conditions.

Due to the rapid development, we were unable to record all the timing of the different stages of development of the zygote/larva. After about 5–6 hpf, we observed the early trochophore stage (Figure 5e), which Trigos et al. (2018) observed after about 22 hpf but at a temperature maintained constant at 21°C. The same difference in development speed was also observed at the late trochophore and early veliger stages (Table 3).

Although we were not able to measure the size of the larvae at the different development stages, the measurements taken do not differ much from those reported by Trigos et al. (2018; Table 3).

The larvae, at about 8–9 dpf, were almost all distributed on the bottom of the tank and moved slowly. After the 9th day, we found only empty shells on the bottom of the tanks.

### 3.4 Molecular Diagnostic Analyses of Pathogens in *P. nobilis*

Neither the larvae nor the filter samples analyzed by standard PCR and qPCR displayed any significant bands on electrophoresis gels. Similarly, no threshold cycle (Ct) values at the qPCR suggested the occurrence of *H. pinnae*.

At the first survey, standard PCR revealed the presence of *H. pinnae* in 6 out of the 11 (54.5% of the total) individuals of *P. nobilis* which were analyzed. Among the six positive samples, three individuals were recently dead, while three were still alive. Five samples were classified as negative for the presence of *H. pinnae* when two attempts to amplify the same sample failed to produce any PCR electrophoretic bands, and Ct values at the qPCR were greatly above 30.

Notably, among the six individuals which tested positive for the standard PCR, the three deceased specimens displayed low Ct values (greatly below 30) at the qPCR suggesting high levels of infection. In contrast, the three still alive individuals displayed just moderately high Ct values at the qPCR (slightly below or equal to 30), suggesting low traces of *H. pinnae* in the samples which could be due to either a weak infection of the tissue or environmental water contamination.

At the second survey (performed after 30 days), analyses were repeated for eight individuals, five of them were surviving and three newly dead. These latter three newly dead individuals were those which resulted positive, but still alive, at the first survey. These individuals again tested positive for *H. pinnae* at the standard PCR during the second survey, and the qPCR showed a relevant decrease in the Ct value compared to the first survey. Meanwhile, the last five surviving individuals tested negative at the standard PCR and displayed high Ct values (again greatly above 30) at the qPCR.

The association between qPCR Ct values lower than 30 and the individual's death was assessed using Pearson's correlation test, which revealed a strong positive association (data not shown). Specifically, all individuals who died during the rounds of surveys tended to have Ct values lower than 30 (in one case equal to 30), while all survivors had higher Ct values always largely above 30, with *R* ^2^ = 1 and a *p*‐value < 0.00001. This indicated a statistically significant association between the two variables (Ct values above or equal to 30 and individual's death). Nevertheless, it is important to note that, although the association is strong, this statistical test does not necessarily imply a direct causal relationship between *H. pinnae* infection levels and death of the fan mussel.

Interestingly, during the 30‐day *H. pinnae* diagnostic surveys in the 11 fan mussels, the same 5 individuals consistently tested negative with standard PCR and showed high Ct values with qPCR. However, one of these individuals died 45 days after the survey's conclusion. Upon examination by new molecular diagnostic analyses, this individual displayed a severe *H. pinnae* infection throughout its organs, suggesting a late‐stage infection that likely developed after the round of diagnostic tests.

## 4 Discussion

Although maintaining pinnids in captivity involves several difficulties, aquaculture could be a valuable tool to restore endangered populations (Acarli 2021; Chávez‐Villalba, Reynaga‐Franco, and Hoyos‐Chairez 2022), through controlled reproduction (Trigos et al. 2018; Hernandis, Prado et al. 2023). Such approach involves appropriate transport and maintenance techniques. Different trials have already been performed to transport adult pinnids in laboratory/aquarium tanks: Trigos and Vicente (2016) transported fun mussels by boat with an average journey time of 15 min. The individuals were immersed in plastic tanks (5 specimens/60 L) and aerated with a portable pump. Prado, Cabanes et al. (2020) transported *Pinna nobilis* juveniles to the laboratory in an aerated cooler. Hernandis (2021) transported the specimens in a van in 300 L tanks, with 10 specimens per tank separated by a plastic net and constant aeration at room temperature. Hernandis et al. (2021) used an “icebox with seawater” to transport *Pinna rudis* specimens.

Also several studies on maintenance of both adult and juvenile specimens of different Pinnidae species in a controlled environment have been performed (Ford and Borrero 2001; Prado et al. 2020; Hernandis 2021), where pathogens are removed through cartridge filtration (10, 5, and 1 μm) combined with sterilizing UV lamps, to ensure the most suitable conditions mirroring those in the environment (such as aeration, photoperiod, water temperature, and food availability; Trigos et al. 2015; Prado, Cabanes et al. 2020; Hernandis 2021; Hernandis et al. 2022).

In order to optimize transport and maintenance protocols for seriously endangered species, the use of model species is instrumental in order to prevent the additional threats. This approach has already been applied in several studies, e.g. *Pinna rudis* , a protected but not endangered species, was used as a model species for the subsequent study of *P. nobilis* (Hernandis et al. 2022). Similarly, but in the case of a gastropod mollusk, *Patella rustica* and *Patella caerulea* were used to test some spawning induction methods and artificial reproduction protocols, later applied to *Patella ferruginea*, a protected and endangered species (Ferranti et al. 2018; Guallart et al. 2020).

In this study, the role of *A. fragilis* was found to be pivotal in facilitating the completion of essential procedures, thereby ensuring the preservation of *P. nobilis* specimens. The cause of the death of the six *A. fragilis* adults, which occurred between May and June (7 months from the transport), is not attributable to transport stress or the transport technique used; in fact, the effects of transport are normally assessed within 15 days following transport (Hernandis et al. 2022).

The transport and maintenance of *A. fragilis* in a controlled environment were proven to be appropriate for drawing up the transport and maintenance protocol for *P. nobilis* specimens. Several methodologies have been tried to transport pinnid specimens, always maintaining the specimens immersed in sea water, at a constant temperature and with aeration (Trigos and Vicente 2016; Prado, Cabanes et al. 2020; Hernandis 2021; Hernandis et al. 2021; Hernandis, Ibarrola et al. 2023). These transport conditions were also considered in this study, and both transport methods tested, vertical and horizontal, proved to be successful. In fact, the survival rate of the specimens in the month following the transport was 100% for *A. fragilis* and 73% for *P. nobilis* (testing positive for *H. pinnae*, the dead specimens).

The maintenance of *P. nobilis* specimens in the laboratory required a greater effort, as in addition to the normal management of water changes, tank cleaning, and food quantity control, it was necessary to sterilize the tank wastewater so as not to contaminate the external environment with pathogens.

During these test phases, working with specimens potentially infected, a control system was carried out through molecular dignostic analyses. These analyses conducted on adult specimens, tanks, filters, and dead larvae, made it possible to simplify and facilitate the management of the *P. nobilis* specimens.

Standard PCR has proven to be a valuable and cost‐effective method for detecting *H. pinnae* infections in *P. nobilis* , achieving positive detection rates corresponding to those obtained with the qPCR. However, qPCR provided a relevant advantage by enabling the monitoring of infection levels in *P. nobilis* tissues through the tracking of Ct value changes over time. This method allowed for the immediate identification of individuals with dangerously increasing infection levels and a high risk of imminent death, as evidenced by a gradual decrease in Ct values.

It is interesting to note that, in the present study, once the infection levels in an individual's mantle begin to produce Ct values at the qPCR below 30, the individual dies within approximately 30 days. The actual existence of this cascade reaction, triggered when Ct values drop below 30 and leading to the rapid animal death, should be further investigated and confirmed through the analysis of numerous cases (if any, hopefully not) in the future. Furthermore, it is important to note that the increased levels of *H. pinnae* infection could merely represent an effective diagnostic factor for the onset and progression of the disease which provokes mass mortality in *P. nobilis* and not necessarily a risk factor associated with it.

Additionally, it is noteworthy that in this study, individuals in contact with sick and dying specimens did not suffer from *H. pinnae* infection and remained healthy. This finding supports earlier research suggesting the existence of *Pinna nobilis* individuals resistant to the mass mortality event (Salis et al. 2022 and references therein). Such resistance could be attributed to genetic introgression with *Pinna rudis* (Vázquez‐Luis et al. 2021) and, in general, could be the product of hanges in genes related to immunity and cellular structure that allowed animals to withstand *H. pinnae* infection. In particular, results of the present study align with Vázquez‐Luis et al. (2021), which report that in some resistant individuals from southern France coast, *H. pinnae* was undetectable by standard PCR, but transcriptomic analysis provided evidence of its RNA, suggesting that these resistant individuals had been exposed to the protozoan without getting seriously infected.

In the future, further analysis on the resistant individuals found in the present study will shed light on the peculiar caractheristics of their genome and gene expression.

With regard to the reproductive phase, better results were obtained with the target *P. nobilis* : As previously documented by Hernandis, Prado et al. (2023), it was not necessary to induce spawning, as the specimens released gametes in response to the stress associated with the transport. The specimens released already fertilized eggs, a phenomenon that has already been observed by other authors (Hernandis, Prado et al. 2023) but in contrast with De Gaulejac (1995), who claimed that *P. nobilis* , as a subsequent hermaphrodite, with asynchronous gametic maturation, avoids self‐fertilization.

The larval development was notably rapid, likely due to the high water temperature (approximately 25°C) during the rearing period. Only the early veliger stage was reached, in contrast to Trigos et al. (2018), who documented the subsequent pediveliger stage. Nevertheless, the information regarding the size of the pediveliger (110 μm) reported by Trigos et al. (2018), is in contrast to that reported by Hashimoto, Ito, and Kanematsu (2023), who state that larvae of the family Pinnidae, including the genera *Atrina* and *Pinna*, can reach a length of 400–800 mm, which is considerably larger than that of other bivalves. Given the possible ubiquity of this trait within the family, it is likely that *P. nobilis* larvae must reach this size in order to successfully complete the settlement and metamorphosis process. In addition to the high temperature, the dose of microalgae was probably too low to support the development and growth of the larvae. We estimate that the cell concentration we provided was 10 times lower than that in Trigos et al. (2018).

The maintenance of the larvae still requires fundamental adjustments, since the results of both Trigos et al. (2018) and the present study provide evidence of unhealthy development. Trigos et al. (2018) reported larvae clusters following the secretion of mucous filaments, possibly as a side effect of the veil loss (possibly a bacterial activity‐related effect) or as a mechanism to improve larval buoyancy and facilitate dispersal. We did not observe any particular behavior of the larvae, except that, at the early trochophore stage, larvae concentrated on the surface in contact with the edge of the beaker and sometimes created “spirals” in the water column. Possibly, the visual lack of mucous filaments in the present study may be attributable to the use of antibiotics in the larval cultures.

The results reported in the present paper represent the first effort to similtaneusly monitor survival, reproduction, and infection states of *P. nobilis* specimens maintained in controlled conditions.

Results indicate that there are still significant knowledge gaps in the succesful long‐term maintenance and larval rearing of these organisms. However, our findings provide a robust baseline for further research that will be conducted in the framework of the Life Pinna and other related and networking projects.

## Author Contributions

**Maria Paola Ferranti:** conceptualization (equal), data curation (equal), formal analysis (equal), investigation (equal), methodology (equal), software (equal), supervision (equal), validation (equal), visualization (equal), writing – original draft (equal), writing – review and editing (equal). **Ilenia Azzena:** conceptualization (equal), data curation (equal), formal analysis (equal), investigation (equal), methodology (equal), writing – original draft (equal), writing – review and editing (equal). **Edoardo Batistini:** conceptualization (equal), investigation (equal), methodology (equal), writing – review and editing (equal). **Daniela Caracciolo:** funding acquisition (equal). **Marco Casu:** conceptualization (equal), data curation (equal), formal analysis (equal), funding acquisition (equal), investigation (equal), methodology (equal), project administration (equal), resources (equal), validation (equal), writing – original draft (equal), writing – review and editing (equal). **Mariachiara Chiantore:** conceptualization (equal), data curation (equal), formal analysis (equal), funding acquisition (equal), investigation (equal), methodology (equal), project administration (equal), resources (equal), supervision (equal), validation (equal), writing – original draft (equal), writing – review and editing (equal). **Saul Ciriaco:** conceptualization (equal), funding acquisition (equal), investigation (equal), methodology (equal), project administration (equal), resources (equal), supervision (equal), validation (equal), writing – review and editing (equal). **Valerio Firpo:** investigation (equal). **Luca Intini:** conceptualization (equal), investigation (equal), writing – original draft (equal), writing – review and editing (equal). **Chiara Locci:** conceptualization (equal), data curation (equal), formal analysis (equal), investigation (equal), methodology (equal), writing – original draft (equal), writing – review and editing (equal). **Monica Montefalcone:** funding acquisition (equal), project administration (equal), resources (equal), writing – review and editing (equal). **Alice Oprandi:** conceptualization (equal), investigation (equal), methodology (equal), visualization (equal), writing – review and editing (equal). **Daria Sanna:** conceptualization (equal), data curation (equal), formal analysis (equal), funding acquisition (equal), investigation (equal), methodology (equal), project administration (equal), resources (equal), supervision (equal), validation (equal), writing – original draft (equal), writing – review and editing (equal). **Fabio Scarpa:** conceptualization (equal), data curation (equal), formal analysis (equal), investigation (equal), methodology (equal), supervision (equal), validation (equal), writing – original draft (equal), writing – review and editing (equal). **Marco Segarich:** conceptualization (equal), data curation (equal), investigation (equal), methodology (equal), supervision (equal), validation (equal), writing – original draft (equal), writing – review and editing (equal).

## Ethics Statement

The Ministry of the Environment, following a positive opinion from ISPRA (Higher Institute for Environmental Protection and Research), has issued a specific authorization required for the collection of species protected by the Habitat Directive.

## Conflicts of Interest

The authors declare no conflicts of interest.

## Supporting information


Data S1.

## Data Availability

All authors make their data available. These data are provided as Data S1 for review and publication.
