# Correction to: “Handling of the Bivalve *Pinna nobilis*, Endangered and Pathogen Affected Species, for Controlled Reproduction: Precautions Taken”

**DOI:** 10.1002/ece3.70932

**Published:** 2025-01-30

Ferranti, M. P., I. Azzena, E. Batistini, D. Caracciolo, M. Casu, M. Chiantore, S. Ciriaco, V. Firpo, L. Intini, C. Locci, M. Montefalcone, A. Oprandi, D. Sanna, F. Scarpa, and M. Segarich. 2024. “Handling of the Bivalve *Pinna nobilis*, Endangered and Pathogen‐Affected Species, for Controlled Reproduction: Precautions Taken.” *Ecology and Evolution* 14, e70565. https://doi.org/10.1002/ece3.70565.

In the “Discussion” section on page 10 (in the second column of the tenth line of the second paragraph), the unit of measurement of “400–800 mm” is incorrect, to be corrected with “400–800 μm.”

We apologize for this error.

